# Individual and seasonal variation in contact rate, connectivity and centrality in red fox (*Vulpes vulpes*) social groups

**DOI:** 10.1038/s41598-019-56713-3

**Published:** 2019-12-27

**Authors:** Jo Dorning, Stephen Harris

**Affiliations:** School of Biological Sciences, University of Bristol, Life Sciences Building, 24 Tyndall Avenue, Bristol, BS8 1TQ UK

**Keywords:** Animal behaviour, Behavioural ecology, Urban ecology

## Abstract

Examining temporal variation in social connectivity and relating this to individual behaviour can help understand the role of individuals within social groups. Although red foxes are solitary foragers, they form social groups at higher population densities. To investigate the effects of season, sex and status on social connectivity in red fox social groups, we set camera traps for four consecutive seasons to record contact rates and social network metrics at food patches in seven fox territories. Higher food availability was associated with higher contact rates. Individual contact rates peaked in different seasons depending on sex and social status. Dominant foxes held central network positions in all seasons but there was no consistent pattern in social connections within territories, suggesting a greater influence of features such as group size and composition on network structure than seasonal behaviour. Increased extraterritorial movements by males during winter contributed to a significant reduction in local network connectivity. Female network strength did not vary with season, suggesting that females play an important role in maintaining year-round group cohesion. These data advance our understanding of canid social systems, the benefits of group-living in solitary foraging carnivores, and the impacts of management interventions for an ecologically important species.

## Introduction

There is a long-standing perception that canids are some of the more social mammals, with social groups based around a dominant pair. However, reviews of canid sociality have focussed on the evolution of group living in canids rather than the social relationships within groups^1–3^. While the social behaviour of some of the larger canids is relatively well-studied, we know little about group structure and stability in the smaller species, probably because their elusive and largely nocturnal behaviour makes them particularly challenging to study.

Red foxes *Vulpes vulpes* are medium-sized canids whose social organisation is determined by population density^4^. At high densities they can form groups of up to ten adults (animals > 1 year old) comprising equal numbers of males and females^4–7^. Radio-tracking data showed that fox intra- and inter-group encounter (association/contact) rates vary with sex and social status, but small sample sizes precluded robust statistical comparisons^8–10^. Fox groups are matrilineal^4^ and, as females receive more affiliative behaviour^11^, they may play an important role in maintaining social group cohesion, although the effect of social status on red fox contact rates is unknown^12^. Preliminary evidence suggests that subordinates may have higher contact rates than dominants^9^. However, associations occur year-round and their patterning and intensity vary seasonally, with generally higher rates of association in summer during cooperative cub rearing and lower rates in winter due to more frequent extraterritorial movements, particularly by males^8^.

The lack of data on the social systems of red foxes is surprising since they are an ecologically and economically important as an invasive species, predator, vector of disease, and in driving ecosystems in much of Australia, Europe and North America, and parts of Arabia and Asia^9,12–14^. The number and strength of an individual’s connections relative to its conspecifics determine its position within a social network. Individuals with more central positions commonly experience increased fitness^15–17^ and may function as ‘keystone individuals’, having a disproportionately large influence on group dynamics and function^18^. While individual relationships shape the structure of the wider network^19,20^, an animal’s position within the network varies with life history patterns. More cohesive groups are more strongly and evenly connected, which provides greater resilience to removal or disruption^21^, and increased connectivity with conspecifics can reduce predation risk^22^ and facilitate transmission of information^23^ and disease^24^.

Understanding the structure of red fox social groups, and their resilience to perturbation, is key to implementing effective management interventions. Previous studies of canid social behaviour relied primarily on demographic data, genetics, proximity loggers or radio-tracking e.g.^25,26^. While proximity loggers and radio-tracking enable continuous monitoring of individuals in both space and time, they underestimate contact rates when fitted to a limited number of individuals^8^, and we could not catch all the animals in our study population^27^. Therefore, we used camera traps set at food patches to investigate whether red fox social cohesion varied between seasons, the effects of individual and environmental attributes on sociality, and whether the structure of red fox social groups is similar to other canids. Foxes are solitary foragers, and so the way they share resources is fundamental to understanding how, and under what circumstances, they form social groups. In particular we addressed the following hypotheses: (i) social cohesion is highest in summer during cub rearing and lowest in winter; (ii) seasons affect males and females differently due to male-biased dispersal and mate-searching behaviour; (iii) dominant foxes occupy more central network positions than subordinates; (iv) females occupy more central network positions due to frequent extra-territorial movements by males; and (v) sociality is correlated with food availability because, while foxes forage alone, they share food patches.

## Results

The structure of networks that included foxes seen on ≥5 days varied greatly between territories and seasons in terms of assortment, individual network position and connectivity.

### Assortment

All networks were disassorted, indicating strong mixing between sexes and statuses, but disassortment was only significant for sex for 4 surveys and for status in 2 surveys (Supplementary Table S1).

### Daily contact rate

**Figure 1:**
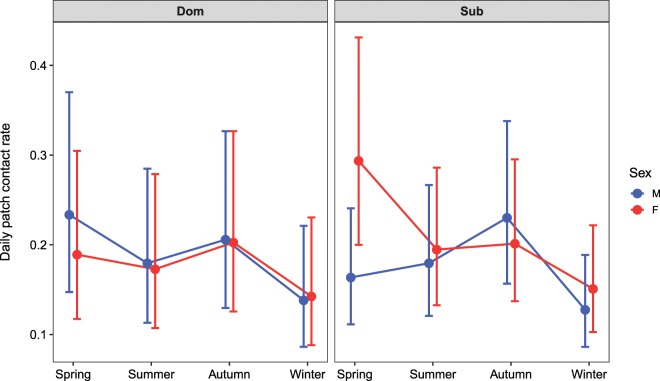
Predicted daily contact rates for resident foxes at food patches, based on a Poisson GLMM (Supplementary Table S2). N = 17,915 observations. Error bars show the 95% confidence intervals. M = male, F = female, Dom = dominant, Sub = subordinate.

We used the gambit of the group approach to define a contact: see Methods. The large standard deviations for the individual and patch random effects in the GLMM indicated high variation in individual contact rates (Supplementary Fig. S1, Supplementary Table S2). The effect of season on daily contact rates at patches depended on sex and social status (GLMM: χ^2^(15) = 241.37, P < 0.001, Fig. 1, Supplementary Table S2). Contact rates were lowest in winter (mean range = 0.128–0.151, Supplementary Table S3) and this was significant for all sex-status combinations when compared to spring (P < 0.05 for all Tukey contrasts, Supplementary Table S3) and autumn (P < 0.001 for all Tukey contrasts), but only for subordinates when compared to summer (P < 0.01 for both Tukey contrasts). Contact rates were highest in spring for subordinate females (mean = 0.294, 95% CI = 0.200–0.431; Tukey contrasts, SP-SU: z-ratio = 6.217, P < 0.001, SP-AU: z-ratio = 5.965, P < 0.001, SP-WI: z-ratio = 10.176, P < 0.001) and dominant males (mean = 0.234, 95% CI = 0.147–0.370; Tukey contrasts, SP-SU: z-ratio = 3.501, P = 0.022, SP-WI: z-ratio = 6.082, P < 0.001). Conversely, contact rates were highest in autumn for subordinate males (mean = 0.230, 95% CI = 0.157–0.338; Tukey contrasts, AU-SP: z-ratio = 5.438, P < 0.001, AU-SU: z-ratio = 3.246, P = 0.055, AU-WI: z-ratio = 8.249, P < 0.001) and dominant females (mean = 0.202, 95% CI = 0.126–0.326; Tukey contrast, AU-WI: z-ratio = 4.468, P < 0.001). Subordinate females had significantly higher contact rates than subordinate males in spring (Tukey contrast: z-ratio = 3.296, P < 0.046), but there were no other differences between sex and status types.

**Figure 2:**
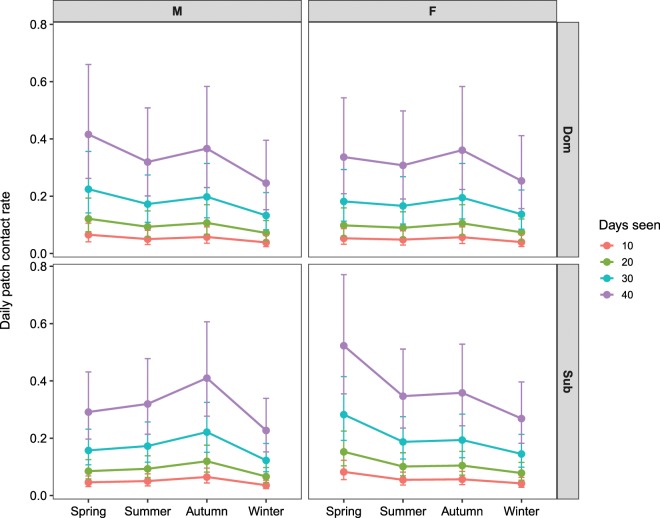
Predicted daily contact rates at patches for foxes with different sighting frequencies in each survey period. Predictions are based on a Poisson GLMM (Supplementary Table S2). N = 17,915 observations. Error bars show 95% confidence intervals. M = male, F = female, Dom = dominant, Sub = subordinate.

Foxes with higher sighting frequencies had higher daily contact rates (χ^2^(1) = 594.05, P < 0.001, Supplementary Table S2) i.e. foxes seen in foraging patches less often were more likely to be observed alone. Predicted contact rates for foxes seen on 10, 20, 30 and 40 days showed an exponential increase in contact rate with sighting frequency (Fig. 2). Based on the GLMM, foxes seen in a patch on 10 days are predicted to associate at that patch 0.052 times/day (95% CI = 0.037–0.073), which extrapolates to one association every 14–27 days, whereas foxes seen in a patch on 40 days will associate at that patch 0.33 times/day (95% CI = 0.237–0.459), which extrapolates to one association every 2–4 days. When data from all territories were pooled, foxes visited on average 2.5 monitored patches/day (SD = 1.2), suggesting that foxes seen daily encountered a conspecific 1–1.5 times/day at food patches.

**Figure 3:**
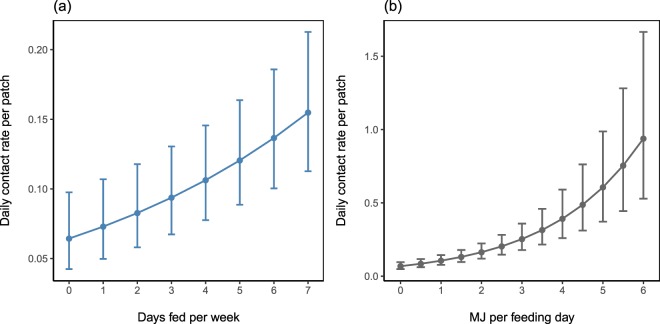
Predicted daily contact rates at patches with increasing food availability, in terms of (**a**) provisioning frequency and (**b**) energy value supplied per feeding day, in megajoules (MJ). N = 17,915 observations.

**Figure 4:**
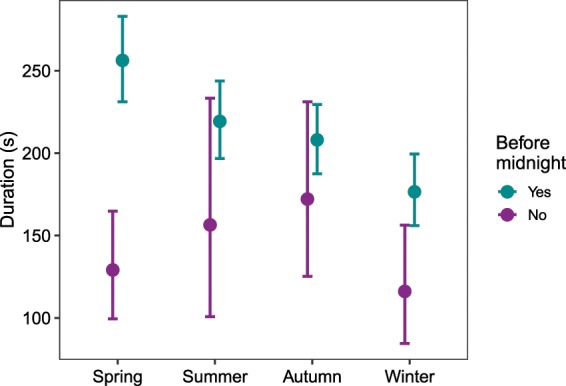
Predicted encounter durations based on 10,000 simulations of the hurdle GLMM in Supplementary Table S5. N = 4015 observations.

Contact rates were significantly higher at patches with higher food availability (Supplementary Table S4) in terms of both provisioning frequency (Fig. 3a) and energy value (Fig. 3b). We recorded 4015 encounters between foxes ≥5 months old: duration of encounters ranged from 0 (encounters where only the start time was known) to 3456 seconds (median 101 seconds, mean 234 seconds). Encounters were significantly longer before midnight than after, particularly during spring (Fig. 4, Supplementary Table S5).

### Global network connectivity

Unweighted density ranged between networks from 0.11–1 (mean = 0.50, SD = 0.27, Supplementary Table S6), indicating that networks contained 11–100% of possible ties. Weighted density, i.e. mean association strength, ranged from 0.02–0.59 (mean = 0.17, SD = 0.13, Supplementary Table S6) and transitivity ranged from 0.34–1 (mean = 0.73, SD = 0.2, Supplementary Table S6). Plotting measures of global connectivity for each territory in the order of data collection demonstrated substantial differences in how networks changed over time (Supplementary Fig. S2). There was no clear seasonal pattern in transitivity or unweighted density, which both changed between seasons in all territories, albeit at different rates. The LMM suggested a slight reduction in unweighted density in autumn compared to spring (β_obs_ = −0.063, P_rand_ = 0.05, Supplementary Table S7), but *post hoc* comparisons revealed no significant differences between seasons. Nor was there any significant seasonal variation in transitivity (Supplementary Table S8). Weighted density was lowest in winter in most territories but this was not significant (Supplementary Table S9). 16–42% of the variation in the measures of global connectivity was attributable to territory identity but the majority of the variation was within territories (Supplementary Tables S7–S9).

The 24 networks with non-random associations in the standardised dataset contained 174 individuals, of which 51 (26♂, 25♀) were categorised as territory residents (Supplementary Table S10). Two further individuals (1♂, 1♀) were only resident in networks with random associations and so were excluded from analyses. Non-residents were from neighbouring territories (51%), previous group members (6%), or strangers i.e. of unknown origin (44%)^7^. They were more often males than females in winter (Wilcoxon signed-rank test: W = 0, P = 0.022, r = −0.824), but there was no significant sex difference in other seasons (spring: W = 19, P = 0.518, r = −0.173; summer: W = 25, P = 0.281, r = −0.288; autumn: W = 17, P = 0.363, r = −0.243).

**Figure 5:**
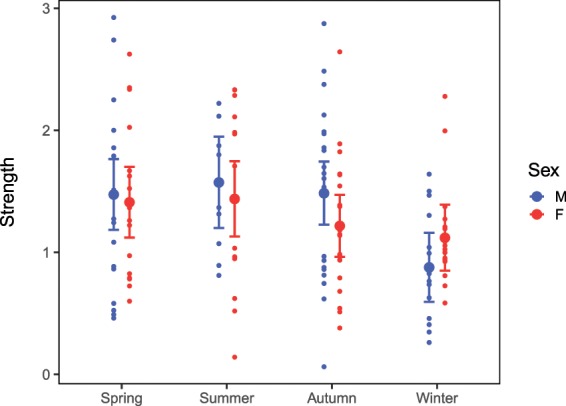
Mean network strength of resident foxes in networks with non-random associations based on an LMM illustrating the interaction effect of sex and season. N = 51 individual foxes. Points show the raw data and error bars show 95% confidence intervals.

Time of day and hence food availability had no significant effect on the network strength of resident foxes (β_obs_ = 0.139, P_rand_ = 0.797). The effect of sex on network strength depended on season (Fig. 5, Supplementary Table S11). Females had higher network strength than males in winter (estimated difference = 0.243, P_rand_ = 0.015) but there were no significant sex differences in other seasons. After correcting P-values for multiple comparisons, network strength did not vary between seasons for females, though there was a tendency for higher network strength in spring compared to autumn (estimated difference = 0.194, P_rand_ = 0.07). Network strength of males was significantly lower in winter compared to summer (estimated difference = 0.696, P_rand_ < 0.001).

Dominant animals had higher network strength than subordinates (β_obs_ = 0.09, P_rand_ < 0.001; the two-tailed P_rand_ was converted to one-tailed by deducting it from 1; Supplementary Fig. S3, Supplementary Table S11) and this effect did not vary with season (Supplementary Table S12). 29% of the variation in network strength was explained by individual ID, and none by territory.

### Centrality measures

The effects of sex and social status on eigenvector centrality had no significant interaction with season, so a reduced model was fitted without season. Sex and status still had no significant effect in the reduced model: 39% of the variance in eigenvector centrality was explained by individual ID and 11% by territory.

**Figure 6:**
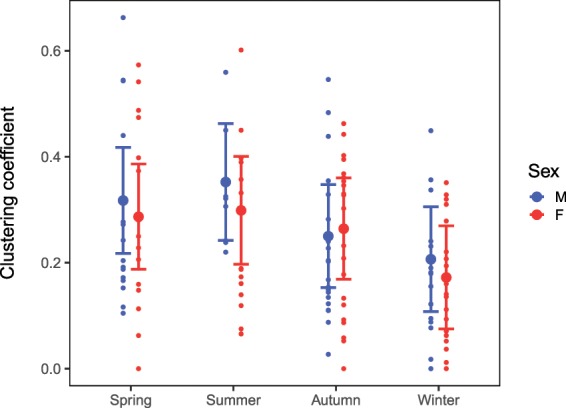
Mean clustering coefficient based on an LMM for male and female resident foxes in networks with non-random associations in different seasons. N = 51 individual foxes. Points show the raw data and error bars 95% confidence intervals.

Sex had a significant effect on clustering coefficient and the nature of this effect depended on season (Fig. 6, Supplementary Table S13). Before P_rand_ values were corrected for multiple testing, males had significantly higher clustering coefficients than females in spring (estimated difference = 0.031, Supplementary Table S14) and summer (estimated difference = 0.054), and females had higher clustering coefficients than males in autumn (estimated difference = 0.014). However, these estimated sex differences were negligible and Holm-adjusted P_rand_ values indicated that they were non-significant.

For males, clustering coefficients were higher in spring and summer compared to autumn and winter. For females, clustering coefficients only differed between autumn and winter (estimated difference = 0.092, P_rand_ < 0.001): the large difference between spring and winter was non-significant (estimated difference = 0.115, P_rand_ = 0.1). There was no detectable relationship between clustering coefficient and social status. Much of the variation in clustering coefficient was explained by territory (42%), while individual ID explained just 2% (Supplementary Table S13).

All three centrality measures showed low repeatability between seasons and no ICCs were significantly different from random (Supplementary Table S15). This is supported by the high residual variation and low proportion of variance explained by individual ID in all models, indicating that variance in centrality was greater within than between individuals.

## Discussion

Our data provide the first quantified analysis of contact rates across all independent individuals (i.e. ≥5 months old) in a wild canid population, albeit that they were limited to high-quality food patches. Camera traps can only monitor a limited number and type of locations, and we were unable to compare data from different habitats or activities. For instance, red foxes typically rest in heavily vegetated areas^28^ that are unsuitable for camera traps, and most resting sites are used for <10 days^10^. Since social networks are shaped by many factors, including behaviour and sampling biases^29^, data from habitats where foxes undertake different activities will further our understanding of canid social behaviour.

However, different techniques do not provide comparable measures of social contact. While radio-tracking/GPS collars and proximity loggers can be used to measure social contacts, a high proportion of the animals we recorded at food patches were not territory residents: radio-tracking/GPS collars or proximity loggers fitted to territory residents would have missed all of these contacts, and so would have failed to identify the role of non-residents in fox social systems. Furthermore, proximity loggers have a limited range, so many encounters will not be recorded, even though they may be socially important. If, as here, the behaviour of the study animals is not affected by camera traps, they have the benefit of monitoring all individuals, not just territory residents, although they may have underestimated those encounters where one fox tried to avoid another and so left the vicinity of the food patch before being photographed. On the positive side, camera traps provide valuable additional data, which enabled us to establish the social status and residency of each fox. However, being an instantaneous record, it was not possible to interpret behaviour in many photographs. For instance, if two foxes were photographed standing in close proximity, it was impossible to determine whether the contact was neutral or the photograph showed an interlude during amicable or antagonistic interactions.

We used temporally overlapping visits to determine when two foxes visited a food patch concurrently: this avoided potential biases due to foxes not being within the field of view of the camera. In the time scale we used to define a contact, foxes would have been aware of each other’s presence: in addition to any interactions recorded by the camera trap, they may have interacted directly out of view of the camera or indirectly using auditory and/or olfactory cues.

The lack of quantified data on red fox behaviour is reflected in the diversity of terms used to describe their social systems. These include primitively social^30^, solitary^31^, spatial groups^32^, showing widespread intraspecific tolerance^33^, socially monogamous^6^, facultatively social^4^, and simple and unsophisticated^34^; whether these terms reflect actual differences in fox social systems is unclear. Perhaps the most common notion is that sociality in red foxes is limited to raising cubs^33^. However, solitary foraging does not mean that red foxes do not have a complex social system: they defend common territories^4,6^, share food patches^35^, have a dominance hierarchy that allows the monopolization of resources^35^, and have long-term social relationships indicative of stable group membership throughout the year^7,36^, all of which suggest that their social system is fundamentally the same as other group-living canids.

Foxes encountered another fox (resident or non-resident) in a given patch at most once every two days, and visited an average of 2.5 patches/day, so we estimate that an individual has 1–1.5 contacts/day at foraging patches. However, this is a minimum estimate because we only monitored food patches where foxes were provisioned regularly. While other food patches were visited less frequently, and so contact rates were likely to be lower, they would still have enhanced the estimates given here. Even so, the minimum rate of contacts we calculated at food patches was similar to an earlier estimate from the same study area of 2.1 intragroup contacts/day (plus 0.1–0.8 intergroup contacts/day depending on season)^8^, albeit that this estimate was based on radio-tracking when group sizes were smaller and data collection was not restricted to food patches.

While patchy resources may act as a catalyst for sociality^37^, foxes probably visited food patches to feed rather than socialise, since contact rates were higher at patches with greater food availability, and longer when food was more likely to be present, suggesting that foxes aggregate at productive foraging patches. However, we have no evidence that social interactions were motivated by food shortage e.g. unlike badgers (*Meles meles*)^38^, fox body weight did not vary with population density. Furthermore, individual network strength did not decline when food was unavailable, indicating that spatiotemporal associations were not simply a by-product of parallel feeding. While they were not suitable for analysis, we recorded a diversity of behavioural interactions at food patches and foxes had preferred and avoided companions, both short and long term^36^. Thus sociality was facilitated by patchy resources rather than foxes simply congregating at productive food patches. While our study was undertaken at high-quality provisioned food patches in an urban area, foxes aggregate at productive food patches in a variety of other habitats, and social interactions similar to the ones we recorded may occur in other habitats.

We expected group connectivity to peak in summer during cooperative cub rearing, but there was little supporting evidence. In most territories, global network connectivity varied widely between seasons but with no consistent pattern, suggesting that seasonal changes in network density and transitivity were not homogeneous across territories^39^. This suggests that they were influenced more by social environment than seasonal behaviour, probably in part due to differences in network size, which ranged from 4 to 16 individuals ≥5 months old (mean 6.9, Supplementary Table S5). Network structure is governed by a multitude of influencing factors that can result in an unpredictable pattern of relationships^40^.

The inconsistent variation in global connectivity may be linked to the lack of between-season repeatability in individual network position which depends on the positions of many other individuals, and so is influenced by network size and structure^16^. This is probably reflected in group composition, as seasons affected the social behaviour of foxes differently depending on their individual attributes. Consistent within-season lagged association rates^36^ suggest that fox network positions were probably repeatable over shorter time periods, but we did not get enough associations/day to make daily or weekly comparisons. Seasonal variation in network centrality may reflect seasonal differences in the roles of individuals^17^, but the lack of consistency also demonstrates a flexibility that may facilitate adaptation to new situations.

Unlike global connectivity, local connectivity showed a clearer seasonal pattern. Individual contact rates were lowest in winter, in contrast to radio-tracking studies which found no seasonal variation in intragroup contact rates and increased intergroup contact rates in winter^8,10^: this difference may be attributable to methodology. Although contact rates declined in the food patches we monitored in winter, they may increase elsewhere in the territory, particularly since males reduce their foraging effort in winter^35^.

Foxes also had lower local clustering coefficients in winter, indicating fewer or weaker connections between associates. As males were least gregarious in winter, the observed reduction in local cliquishness alongside lower contact rates may be explained by increased extraterritorial movement by dispersing and mate-seeking males^7^. In contrast, home-range overlap increases intergroup encounter rates^8,41^, facilitating the formation of temporary and weak social connections with non-resident foxes, whereas reduced time on the home territory may weaken social connections between residents^42^. Lower contact rates, but with a wider variety of individuals, resulted in more sparsely connected networks, and reduced group cohesion. Although direct contacts play an important role in maintaining social cohesion^8^, foxes also maintain social contact through scent marking and/or vocalisations. Females also had lower contact rates and clustering coefficients in winter, but showed no seasonal variation in network strength: when male network strength dropped in winter, females occupied more central network positions and so may play an important role in maintaining group connectivity while males are elsewhere.

Male local clustering coefficients were significantly higher in summer compared to winter, and higher than females in spring and summer. While the significance of these sex differences did not withstand correction for multiple testing, it may be better to rely on effect size rather than P-values in network analysis^17^. Nodes with a high clustering coefficient are important for group stability and cause greater disruption to the network following their removal^21^. Since all foxes had higher clustering coefficients in spring, summer and autumn, removing a key individual during these seasons may cause greater social disruption than during winter.

Individual contact rates peaked in different seasons depending on sex and social status. In spring, higher contact rates for dominant males may help repair social bonds weakened over winter. Subordinate females may attempt to build stronger relationships in spring prior to giving birth to reduce the risk of infanticide. Alternatively, they may simply encounter more individuals when providing alloparental care, though subordinate foxes of both sexes provision cubs^43^. Higher autumn contact rates of subordinate males may be due to dispersers establishing social bonds with potential new groups, or philopatric individuals reinforcing social bonds within their natal group, as contact rates of dominant females also peaked in autumn.

Dominant foxes had higher network strength than subordinates throughout the year, but status did not influence eigenvector centrality or clustering coefficient, indicating that dominants had more central network positions with respect to direct, but not indirect, connectivity, supporting the general assumption that the breeding pair forms the core social unit in group-living canids. Dominance is commonly linked to increased strength in social networks^40^, which brings fitness benefits such as decreased parasite burden^44^, faster access to information about food resources^45^, and ultimately increased reproductive output and survival^46^. For foxes, stronger social connections facilitate familiarity between territory residents and thereby improve foraging success: less aggression at shared patches may increase foraging rate, and discriminating between familiar and unfamiliar conspecifics allows the use of reliable information from residents^47^. For dominant animals, higher network strength may also reinforce their social rank^20^ and promote cooperation e.g. during cub rearing, as alloparental care by subordinates reduces provisioning effort by dominant foxes^43^.

Between-individual variation in eigenvector centrality was not explained by social status or sex, indicating the influence of individual attributes such as age that we could not measure. Fox home ranges increase with age in their first year^48^, which may also increase indirect connectivity, so season may influence patterns of association differently for adults (≥12 months) and animals ≥5 to 12 months old. Poor body condition or infection with diseases such as sarcoptic mange (*Sarcoptes scabiei*) also alters behaviour in foxes^28^, and so may lead to reduced direct and/or indirect social connectivity^49^. It is therefore important to advance our understanding of the effects of disease on fox social networks to improve models of disease spread both within and between fox social groups.

Eigenvector centrality is sensitive to missing data in small networks and so it is possible that, by analysing territories separately and filtering out foxes seen on <5 days, we may have hidden possible ‘brokers’ that linked networks in different territories^50^. Alternatively, eigenvector centrality may simply be less relevant for territorial species living in relatively closed groups, where individuals are mostly linked by direct, rather than indirect, social connections^51^.

## Conclusions

How solitary-foraging group-living carnivores share key resources is fundamental to understanding how and under what circumstances they form social groups: our data advance understanding of the structure of canid social systems and the costs and benefits of group-living in solitary foraging carnivores generally. Recent studies have shown that the loss of some group members can have a disproportionate, and long-lasting, impact on canid group structure and stability^4,52^. Using social networks to analyse how canid social groups operate and the role of different individuals in maintaining group cohesion will improve strategies for disease and population control, conservation management, and for assessing the resilience of canids to population perturbation.

## Methods

### Study area and data collection

We conducted camera-trap surveys between July 2013 and June 2015 in seven fox territories in the city of Bristol, UK^4,10,25,53^. We selected four to six back gardens (food patches)/territory where householders provisioned foxes ≥2 days/week^35,36^. Cameras were active continuously for 40 days for four consecutive seasons: spring (March-May; birth, early cub rearing), summer (June-August; late cub rearing/early independence), autumn (September-November; onset of dispersal), winter (December-February; peak dispersal/mating). The first survey on each territory varied; consecutive surveys were ≥39 days apart^35,54^. Since foxes are crepuscular/nocturnal^55^, ‘days’ started/ended at noon to ensure independent sampling.

We excluded foxes <5 months old because they were mainly active around their natal den^48^. Where possible, sex was determined from visible features. Foxes were identified in 99% of photographs^54^. A fox was only considered resident on one territory/season: residents were photographed on ≥20 days/season and shared ≥2 associations with another resident^7,33^. The dominant male and female elicited submissive postures from other (subordinate) group members^25^.

Interactions can only be inferred from photographs, so we defined social encounters as associations, using the gambit of the group approach^56^. Intervals ≥15 mins between photographs of a fox at a food patch indicated separate visits^35^: overlapping visits identified two foxes visiting a food patch concurrently. We quantified social connectivity using individual contact rates and global and individual network metrics from 28 social networks (one/group/survey). Contact rates were calculated for foxes seen ≥5 days/season in any patch: this reduced noise from seldom-observed individuals^37^. Not all patches were included every season^7,36^, so network metrics were calculated for foxes seen ≥5 days in a standardised dataset using the same number of patches/season to ensure comparability within a territory: fox community structure matches territorial space use^36^.

We used R version 3.3.0 unless otherwise stated. Mixed models accounted for repeated measures, fitted by maximum likelihood using ‘*lme4*’ version 1.1–11^57^ and simplified using stepwise reduction with deviance testing.

### Daily contact rate

This was the mean number of associations an individual had with any other fox/patch/day. We used a Poisson GLMM (generalised linear mixed model) to investigate whether individual attributes and season influenced daily contact rates. Fixed effects were sighting frequency (number of days seen/patch/survey) and a three-way interaction between sex, social status and season. Random effects were individual, patch ID and their interactions, as patch effects were expected to vary between individuals^35^. Residual plots confirmed the final model fit was satisfactory and residuals were not over-dispersed. We examined the interaction using *post hoc* Tukey tests in ‘*lsmeans*’ version 2.23^58^ and adjusted P-values for multiple testing using the Šidák correction.

We fitted a separate Poisson GLMM with the same random effect structure to test whether provisioning frequency and energy value affected both frequency and length of contact rates^35^. Householders recorded daily provisioning time, type and quantity of food. Provisioning frequency was the mean number of days/week food was provided; energy value was the mean nutritional value (MJ) of food supplied/day^55^. They had a moderate positive correlation (r_s_ = 0.45), but variance inflation factors in the model confirmed independence^59^.

### Encounter duration

This was the total overlap between recorded visits by two individuals e.g. if A visited from 20:00–20:30 and B from 20:20–20:30, encounter duration was 600 seconds. Householders provided food in the afternoon/evening. Since it was generally consumed by midnight, we split ‘days’ into before (food) and after midnight (no food) and modelled the relationship between encounter duration in seconds and food availability using the ‘before midnight’ boolean proxy. We set durations to zero for encounters where only the start time was known. As 2899/4015 observed encounters were ‘0 seconds’ long, we used ‘*GLMMadaptive*’ version 0.6–5^60^ in R version 3.6.0 to fit a negative binomial mixed effects hurdle model: a logistic regression differentiated between zero and non-zero durations, and a linear regression modelled durations of positive length. Both parts of the maximal model had a fixed effect for the interaction ‘before midnight’ and season, and dyad ID as the random effect. We used stepwise simplification with deviance tests to identify the minimal model.

### Network construction and statistical processes

SOCPROG v.2.6 was used to construct weighted association matrices based on the simple ratio index and one-day sampling periods^61^: association matrices were exported to R. All analyses were on weighted networks using mixed models. Since network data are not statistically independent, we calculated P-values for all network measures by comparing observed model coefficients with a distribution of null-model (random) coefficients obtained by fitting the same model data from random networks created by data-stream permutations^17,62,63^. We used ‘*asnipe*’ version 0.91^64^ to randomise associations within territories and days to control for spatiotemporal variation in individual sighting history. Observed coefficients differed statistically from random if they were higher/lower than 95% of the random coefficients^62^. 2000 data stream permutations (10 swaps/permutation) were required to stabilise P-values, calculated as P_rand_ = Σ(β_obs_ < β_rand_)/2000. β was the model coefficient from either observed (β_obs_) or random (β_rand_) networks, 2000 the number of null models. P-values were two-tailed and significant at the 0.05 level when 0.025 > P_rand_ > 0.975.

For model simplification, we compared coefficients from the full model to coefficients from a distribution of null models based on data stream permutations. We removed non-significant terms and re-calculated P-values to report final model coefficients. P-values for *post hoc* tests were generated by comparing observed and random estimated contrast values using ‘*lsmeans*’ version 2.23^58^. *Post hoc* P-values were adjusted for multiple testing^65^, interpreted as one-tailed and significant when P_rand_ < 0.05.

### Global network connectivity

To investigate the effect of season on group cohesion, we calculated unweighted density, weighted density and transitivity of each network using ‘*sna*’ version 2.3–2^66^. Unweighted density is the proportion of possible edges in the network^45^. Weighted density (sum of edge weights/number of possible edges) represents the mean association index^17,66^. High density networks have more and/or stronger connections/node and are more stable (cohesive)^20^. Transitivity is the probability that two associates of a given node are connected^67^ and describes the overall level of (unweighted) clustering in the network^17^ and balance of relationships^19^. Networks with high transitivity (clustering) have strong local, but weaker global, cohesion, so are more susceptible to fragmentation^20^.

We fitted LMMs to each global network measure with season as the fixed effect and territory as the random effect. We used a Gausian LMM for transitivity and unweighted density and a Gamma GLMM with log link for weighted density.

### Assortment

We tested for assortment by sex and social status in each weighted network by calculating the assortativity coefficient (range −1 to 1)^68^ using ‘*assortnet*’^63^; positive values indicate that nodes tend to associate with others of similar phenotypes, negative values that nodes with dissimilar phenotypes are more strongly connected.

### Individual network position

We assessed individual prominence in each network using strength, eigenvector centrality and clustering coefficient. Strength (weighted degree) measures direct connectivity, calculated as the sum of edge weights between a focal node and its immediate neighbours^69^. Individuals with high strength associate with more conspecifics more often, and occupy more central network positions. Eigenvector centrality incorporates the number and weight of direct and indirect social connections^51^ and describes the relative importance of a focal node. Individuals with high eigenvector centrality have high strength and/or are connected to associates with high strength^20^. We calculated strength and eigenvector centrality using ‘*sna*’ version 2.3–2^66^. Clustering coefficients (values 0 to 1) represent local group cohesiveness and describe the proportion of a focal node’s associates that are themselves connected^70^. Groups are more likely to fragment following removal of a node with a high clustering coefficient, so can identify key individuals in maintaining social cohesion^21^. We calculated the weighted clustering coefficient^71^ using ‘*qgraph*’ version 1.3.1^72^: this avoided assigning the same coefficient to individuals with the same number of edges but different edge weights. Clustering coefficient was adjusted for network density within the ‘*qgraph*’ function ‘*clustOnnela*’. We did not normalise strength and eigenvector because networks created with the same techniques are comparable^73^; we used absolute values to compare network position at different times.

LMMs were used to investigate the effect of social (sex, status) and environmental (season, food availability) factors on each network centrality statistic separately. We calculated individual strength before (food) and after (no food) midnight. Foxes only ever seen alone had a strength of zero, so we added 1 to all values and log_10_-transformed them to fit a lognormal LMM. The maximal model included sex, status, season and a boolean for ‘before midnight’. Territory and individual ID were random effects. Since ‘before midnight’ had no effect on strength, we recalculated strength from whole days. This was normally distributed, so the whole-day model was a Gaussian LMM.

We used Gaussian LMMs to investigate whether eigenvector centrality and clustering coefficient were influenced by sex, status and their two-way interactions with season.

We only calculated individual network centrality measures and assortment for territory residents in the 24 networks where foxes associated non-randomly, as determined using the Manly/Bejder test in SOCPROG v2.6^36^.

### Repeatability of network position

Intra-class correlation coefficients (ICC) were used to determine whether individuals had similar network positions in each season. ICC was the proportion of variance explained by individual in a null model fitted for each centrality measure; individual ID and territory were random effects. These null models included data from territory residents in all 28 networks i.e. including those with random associations. ICCs < 0.3 indicate low between-season repeatability, ICCs > 0.7 high repeatability^74^.

### Ethical approval

No foxes were captured or handled for this project. The study was observational and approved by the University of Bristol’s Animal Welfare & Ethical Review Board.

## Supplementary information

Supplementary information.

## Data Availability

Data are available at 10.6084/m9.figshare.9917633.
