# Supplementary material for: Individual and seasonal variation in contact rate, connectivity and centrality in red fox (Vulpes vulpes) social groups
Source: Sci Rep. 2019 Dec 27;9:20095. doi: 10.1038/s41598-019-56713-3 (PMC6934461; doi:10.1038/s41598-019-56713-3)
Supplement: Supplementary file 1 — Supplementary information. [file 41598_2019_56713_MOESM1_ESM.pdf]

# Supplementary information

## Individual and seasonal variation in contact rate, connectivity and centrality in red fox (*Vulpes vulpes*) social groups

Jo Dorning*, Stephen Harris

**Supplementary Table S1.** Assortment coefficients (r) for sex and status for each of the 24 networks included in the analysis. Dashes indicate networks with random associations that were not analysed. Significant P-values are shown in bold.

| Territory | Season | Sex | | | Social status | | |
|---|---|---|---|---|---|---|---|
| | | r | SE | P | r | SE | P |
| T1 | SP | -0.307 | 0.115 | **1.000** | -0.314 | 0.081 | **1.000** |
| T1 | SU | -0.371 | 0.282 | 0.881 | -0.158 | 0.236 | 0.034 |
| T1 | AU | -0.204 | 0.124 | 0.812 | -0.130 | 0.069 | 0.416 |
| T1 | WI | -0.373 | 0.156 | **0.997** | -0.271 | 0.088 | **0.997** |
| T2 | SP | -0.323 | 0.163 | 0.687 | -0.275 | 0.272 | 0.354 |
| T2 | SU | - | - | - | - | - | - |
| T2 | AU | -0.252 | 0.136 | 0.291 | -0.309 | 0.351 | 0.333 |
| T2 | WI | -0.452 | 0.219 | 0.710 | -0.105 | 0.265 | 0.323 |
| T3 | SP | -0.452 | 0.279 | **0.979** | -0.183 | 0.323 | 0.050 |
| T3 | SU | -0.397 | 0.265 | 0.884 | -0.163 | 0.293 | 0.064 |
| T3 | AU | -0.166 | 0.354 | 0.173 | -0.313 | 0.319 | 0.300 |
| T3 | WI | -0.394 | 0.288 | 0.452 | -0.082 | 0.229 | 0.377 |
| T4 | SP | - | - | - | - | - | - |
| T4 | SU | - | - | - | - | - | - |
| T4 | AU | -0.276 | 0.200 | 0.853 | -0.033 | 0.217 | 0.092 |
| T4 | WI | -0.501 | 0.281 | 0.727 | -0.264 | 0.365 | 0.243 |
| T5 | SP | 0.447 | 0.333 | 0.367 | -0.783 | 0.321 | 0.514 |
| T5 | SU | -0.514 | 0.320 | 0.489 | -0.573 | 0.322 | 0.577 |
| T5 | AU | -0.202 | 0.146 | 0.740 | -0.202 | 0.086 | 0.858 |
| T5 | WI | - | - | - | - | - | - |
| T6 | SP | -0.249 | 0.469 | 0.289 | -0.185 | 0.314 | 0.508 |
| T6 | SU | -0.340 | 0.137 | **0.988** | -0.119 | 0.237 | 0.076 |
| T6 | AU | -0.344 | 0.148 | 0.887 | -0.055 | 0.226 | 0.200 |
| T6 | WI | -0.172 | 0.170 | 0.570 | -0.071 | 0.187 | 0.233 |
| T7 | SP | -0.364 | 0.191 | 0.428 | -0.335 | 0.316 | 0.611 |
| T7 | SU | -0.794 | 0.475 | 0.838 | -0.252 | 0.269 | 0.184 |
| T7 | AU | -0.404 | 0.182 | 0.662 | -0.289 | 0.315 | 0.126 |
| T7 | WI | -0.456 | 0.190 | 0.742 | -0.123 | 0.338 | 0.156 |

**Supplementary Table S2.** Poisson GLMM investigating the effect of individual and environmental attributes on daily contact rates at food patches, for foxes seen on ≥ 5 days in any patch and territory. Coefficients (β) and standard errors are on the log scale. Significant P-values are shown in bold. Unit of analysis = number of contacts/individual/patch/day. N = 17,915 observations.

| **Model parameter** | **β** | **SE** | **z** | $\chi^2$ | **df** | **P** |
|---|---|---|---|---|---|---|
| *Fixed effects* | | | | | | |
| Intercept | -3.553 | 0.259 | -13.724 | | | |
| Days seen | 0.062 | 0.003 | 22.697 | 594.05 | 1 | **<0.001** |
| Sex * status * season | | | | 75.284 | 10 | **<0.001** |
| Sex (male) | 0.213 | 0.266 | 0.800 | | | |
| Status (subordinate) | 0.442 | 0.233 | 1.898 | | | |
| Season (summer) | -0.090 | 0.071 | -1.257 | | | |
| Season (autumn) | 0.069 | 0.073 | 0.953 | | | |
| Season (winter) | -0.283 | 0.073 | -3.898 | | | |
| Sex (male) : status (subordinate) | -0.798 | 0.319 | -2.501 | | | |
| Sex (male) : season (summer) | -0.174 | 0.103 | -1.682 | | | |
| Sex (male) : season (autumn) | -0.196 | 0.103 | -1.901 | | | |
| Sex (male) : season (winter) | -0.242 | 0.113 | -2.153 | | | |
| Status (subordinate) : season (summer) | -0.321 | 0.097 | -3.306 | | | |
| Status (subordinate) : season (autumn) | -0.447 | 0.096 | -4.641 | | | |
| Status (subordinate) : season (winter) | -0.383 | 0.098 | -3.922 | | | |
| Sex (male) : status (subordinate) : season (summer) | 0.677 | 0.144 | 4.692 | | | |
| Sex (male) : status (subordinate) : season (autumn) | 0.915 | 0.136 | 6.728 | | | |
| Sex (male) : status (subordinate) : season (winter) | 0.659 | 0.147 | 4.472 | | | |
| | | | | | | |
| *Random effects* | *Variance* | *SD* | *% total* | | | |
| Individual ID (N = 132) | 0.197 | 0.443 | 17 | | | |
| Patch ID (N = 35) | 0.216 | 0.465 | 19 | | | |
| Individual * patch | 0.729 | 0.854 | 64 | | | |

Reference categories were sex = female, social status = dominant, season = spring

**Supplementary Table S3.** Mean daily contact rates at food patches for foxes seen on an average number of days. Estimates are based on a Poisson GLMM and back-transformed from the log scale. Tukey test P-values are from comparisons between seasons.

| Season | Sex | Social status | Mean contact rate | 2.5% CI | 97.5% CI | Tukey test P-value | | |
|---|---|---|---|---|---|---|---|---|
| | | | | | | Summer | Autumn | Winter |
| Spring | M | Dom | 0.234 | 0.147 | 0.370 | **0.022** | 0.985 | **<0.001** |
| | | Sub | 0.164 | 0.111 | 0.241 | 1.000 | **<0.001** | **0.024** |
| | F | Dom | 0.189 | 0.117 | 0.304 | 1.000 | 1.000 | **0.005** |
| | | Sub | 0.294 | 0.200 | 0.431 | **<0.001** | **<0.001** | **<0.001** |
| Summer | M | Dom | 0.180 | 0.113 | 0.285 | | 0.961 | 0.181 |
| | | Sub | 0.179 | 0.121 | 0.267 | | 0.055 | **0.004** |
| | F | Dom | 0.173 | 0.107 | 0.278 | | 0.813 | 0.498 |
| | | Sub | 0.195 | 0.133 | 0.286 | | 1.000 | **0.007** |
| Autumn | M | Dom | 0.206 | 0.130 | 0.327 | | | **<0.001** |
| | | Sub | 0.230 | 0.157 | 0.338 | | | **<0.001** |
| | F | Dom | 0.202 | 0.126 | 0.326 | | | **<0.001** |
| | | Sub | 0.201 | 0.137 | 0.295 | | | **<0.001** |
| Winter | M | Dom | 0.138 | 0.086 | 0.221 | | | |
| | | Sub | 0.128 | 0.086 | 0.189 | | | |
| | F | Dom | 0.142 | 0.088 | 0.230 | | | |
| | | Sub | 0.151 | 0.103 | 0.222 | | | |

M = male, F = female, Dom = dominant, Sub = subordinate

**Supplementary Table S4.** Poisson GLMM investigating the effects of food availability on daily contact rate at food patches for foxes seen on ≥ 5 days in any territory. Coefficients (β) are on the log scale and significant P-values are shown in bold. Unit of analysis = number of contacts/individual/patch/day. N = 17,915 observations.

| **Model parameter** | **β** | **SE** | **z** | $\chi^2$ | **df** | **P** |
|---|---|---|---|---|---|---|
| *Fixed effects* | | | | | | |
| Intercept | -3.376 | 0.219 | -15.388 | | | |
| Days fed/week | 0.125 | 0.027 | 4.697 | 21.857 | 1 | **<0.001** |
| MJ/feeding day | 0.437 | 0.054 | 8.094 | 62.912 | 1 | **<0.001** |
| | | | | | | |
| *Random effects* | *Variance* | *SD* | *% total* | | | |
| Individual ID (N = 132) | 0.440 | 0.663 | 32 | | | |
| Patch ID (N = 35) | 0.416 | 0.645 | 31 | | | |
| Individual * patch | 0.499 | 0.707 | 37 | | | |

MJ = megajoules

**Supplementary Table S5.** Hurdle GLMM investigating the effects of variable 'before midnight', as a proxy for food availability, and its interaction with season on encounter duration for foxes seen on ≥ 5 days in any territory. Significant P-values are shown in bold. The interaction term improved model fit slightly (p=0.052) so was retained in the final model. Unit of analysis = dyadic encounter duration in seconds. N = 4015 observations.

| Model parameter | β | SE | z | $\chi^2$ | df | P |
|---|---|---|---|---|---|---|
| *Fixed effects: higher coefficients mean longer durations* | | | | | | |
| Intercept | 5.266 | 0.122 | 43.343 | | | |
| Season | | | | 24.08 | 6 | **0.001** |
| Season (summer) | 0.151 | 0.237 | 0.638 | | | |
| Season (autumn) | 0.396 | 0.185 | 2.144 | | | |
| Season (winter) | 0.039 | 0.182 | 0.215 | | | |
| Before midnight (yes) | 0.553 | 0.125 | 4.447 | 20.75 | 4 | **<0.001** |
| Season * Before midnight | | | | 7.72 | 3 | **0.052** |
| Season (summer) : before midnight (yes) | -0.321 | 0.243 | -1.323 | | | |
| Season (autumn) : before midnight (yes) | -0.523 | 0.193 | -2.717 | | | |
| Season (winter) : before midnight (yes) | -0.300 | 0.192 | -1.563 | | | |
| *Zero-part coefficients: higher coefficients mean shorter durations* | | | | | | |
| Intercept | -0.681 | 0.142 | -4.807 | | | |
| Season | | | | 23.29 | 3 | **<0.001** |
| Season (summer) | -0.064 | 0.116 | -0.552 | | | |
| Season (autumn) | 0.302 | 0.101 | 2.989 | | | |
| Season (winter) | 0.400 | 0.113 | 3.535 | | | |
| Before midnight (yes) | -0.474 | 0.128 | -3.704 | 13.36 | 1 | **<0.001** |
| *Random effects* | *Std. Dev.* | | | | | |
| Intercept | 0.2505 | | | | | |
| Dyad ID (N = 200) | 0.3952 | | | 39.34 | 2 | **<0.001** |

The reference category for season = spring

**Supplementary Table S6.** Network size (N) and global connectivity based on simple ratio indices. Networks included only foxes seen on ≥ 5 days. The main component excludes isolated individuals and pairs. Weighted density is equivalent to the mean association index. Unweighted density is the proportion of possible existing ties. Transitivity is the proportion of potentially intransitive triads.

| Territory | Season | N (M:F) | N in main component (M:F) | Unweighted density | Weighted density | Transitivity |
|---|---|---|---|---|---|---|
| T1 | Spring | 13 (8:5) | 11 (7:4) | 0.385 | 0.123 | 0.718 |
| | Summer | 7 (2:5) | 7 (2:5) | 0.571 | 0.208 | 0.6 |
| | Autumn | 12 (8:4) | 10 (7:3) | 0.515 | 0.113 | 0.797 |
| | Winter | 13 (8:5) | 13 (8:5) | 0.449 | 0.095 | 0.770 |
| T2 | Spring | 7 (3:4) | 7 (3:4) | 0.524 | 0.150 | 0.636 |
| | Summer | 5 (2:3) | 5 (2:3) | 0.7 | 0.209 | 0.643 |
| | Autumn | 5 (2:3) | 5 (2:3) | 0.7 | 0.208 | 0.8 |
| | Winter | 7 (4:3) | 6 (3:3) | 0.381 | 0.102 | 0.529 |
| T3 | Spring | 7 (3:4) | 6 (3:3) | 0.429 | 0.215 | 0.652 |
| | Summer | 5 (3:2) | 5 (3:2) | 0.9 | 0.416 | 0.875 |
| | Autumn | 7 (3:4) | 6 (3:3) | 0.381 | 0.120 | 0.632 |
| | Winter | 9 (5:4) | 6 (3:3) | 0.250 | 0.070 | 0.6 |
| T4 | Spring | 4 (2:2) | 4 (2:2) | 1 | 0.322 | 1 |
| | Summer | 4 (2:2) | 4 (2:2) | 1 | 0.409 | 1 |
| | Autumn | 10 (5:5) | 6 (3:3) | 0.289 | 0.148 | 0.846 |
| | Winter | 7 (4:3) | 5 (3:2) | 0.333 | 0.085 | 0.8 |
| T5 | Spring | 10 (6:4) | 5 (3:2) | 0.111 | 0.033 | 0.5 |
| | Summer | 9 (5:4) | 5 (3:2) | 0.139 | 0.040 | 0.375 |
| | Autumn | 9 (6:3) | 9 (6:3) | 0.806 | 0.179 | 0.960 |
| | Winter | 13 (8:5) | 10 (7:3) | 0.244 | 0.040 | 0.6 |
| T6 | Spring | 6 (4:2) | 4 (2:2) | 0.4 | 0.112 | 1 |
| | Summer | 12 (6:6) | 9 (5:4) | 0.333 | 0.084 | 0.771 |
| | Autumn | 21 (11:10) | 13 (5:8) | 0.114 | 0.022 | 0.462 |
| | Winter | 21 (11:10) | 16 (8:8) | 0.110 | 0.027 | 0.339 |
| T7 | Spring | 4 (1:3) | 4 (1:3) | 1 | 0.558 | 1 |
| | Summer | 4 (1:3) | 4 (1:3) | 0.667 | 0.258 | 0.6 |
| | Autumn | 5 (1:4) | 4 (1:3) | 0.6 | 0.259 | 1 |
| | Winter | 5 (1:4) | 4 (1:3) | 0.6 | 0.208 | 1 |

M = male, F = female

**Supplementary Table S7.** Linear mixed model (LMM) investigating the effect of season on unweighted density. $P_{rand}$ is the proportion of model coefficients (β) based on randomised data that were greater than those based on observed data. Significant $P_{rand}$ values are shown in bold when 0.025 > $P_{rand}$ > 0.975. Unit of analysis = unweighted density. N = 28 observations.

| **Model parameter** | **β** | **SE** | **df** | **t** | **$P_{rand}$** |
|---|---|---|---|---|---|
| *Fixed effects* | | | | | |
| Intercept | 0.550 | 0.093 | 25 | 5.883 | **1.000** |
| Season (summer) | 0.066 | 0.117 | 21 | 0.564 | 0.149 |
| Season (autumn) | -0.063 | 0.117 | 21 | -0.541 | 0.050 |
| Season (winter) | -0.212 | 0.117 | 21 | -1.808 | 0.087 |
| | | | | | |
| *Random effects* | *Variance* | *SD* | *% total* | | |
| Territory (N = 7) | 0.013 | 0.115 | 22 | | |
| Residual | 0.048 | 0.219 | 78 | | |

The reference category for season = spring

**Supplementary Table S8.** LMM investigating the effect of season on network transitivity. $P_{rand}$ is the proportion of model coefficients (β) based on randomised data that were greater than those based on observed data. Significant $P_{rand}$ values are shown in bold when 0.025 > $P_{rand}$ > 0.975. Unit of analysis = network transitivity. N = 28 observations.

| **Model parameter** | **β** | **SE** | **df** | **t** | **$P_{rand}$** |
|---|---|---|---|---|---|
| *Fixed effects* | | | | | |
| Intercept | 0.787 | 0.072 | 26 | 11.001 | 0.396 |
| Season (summer) | -0.092 | 0.093 | 21 | -0.988 | 0.969 |
| Season (autumn) | -0.001 | 0.093 | 21 | -0.016 | 0.729 |
| Season (winter) | -0.124 | 0.093 | 21 | -1.335 | 0.837 |
| | | | | | |
| *Random effects* | *Variance* | *SD* | *% total* | | |
| Territory (N = 7) | 0.006 | 0.075 | 16 | | |
| Residual | 0.030 | 0.174 | 84 | | |

The reference category for season = spring

**Supplementary Table S9.** Gamma GLMM investigating the effect of season on weighted network density. $P_{rand}$ is the proportion of model coefficients (β) based on randomised data that were greater than those based on observed data. Significant $P_{rand}$ values are shown in bold when 0.025 > $P_{rand}$ > 0.975. Unit of analysis = weighted network density. N = 28 observations.

| **Model parameter** | **β** | **SE** | **t** | **$P_{rand}$** |
|---|---|---|---|---|
| *Fixed effects* | | | | |
| Intercept | -1.748 | 0.300 | -5.835 | **<0.001** |
| Season (summer) | 0.125 | 0.242 | 0.514 | 0.734 |
| Season (autumn) | -0.237 | 0.251 | -0.942 | 0.907 |
| Season (winter) | -0.840 | 0.242 | -3.469 | 0.151 |
| | | | | |
| *Random effects* | *Variance* | *SD* | *% total* | |
| Territory (N = 7) | 0.169 | 0.411 | 42 | |
| Residual | 0.237 | 0.487 | 58 | |

The reference category for season = spring

**Supplementary Table S10.** Number of territory residents in each network included in the statistical models. Dashes indicate networks with random associations that were not used in the models.

| Territory | Season | Dominant | | Subordinate | | Total |
|---|---|---|---|---|---|---|
| | | M | F | M | F | |
| T1 | Spring | 1 | 1 | 6 | 3 | 11 |
| | Summer | 1 | 1 | 1 | 3 | 6 |
| | Autumn | 1 | 1 | 6 | 2 | 10 |
| | Winter | 1 | 1 | 6 | 4 | 12 |
| T2 | Spring | 1 | 1 | 0 | 2 | 4 |
| | Summer | - | - | - | - | 0 |
| | Autumn | 1 | 1 | 0 | 2 | 4 |
| | Winter | 1 | 1 | 1 | 2 | 5 |
| T3 | Spring | 1 | 1 | 2 | 1 | 5 |
| | Summer | 1 | 1 | 2 | 1 | 5 |
| | Autumn | 1 | 1 | 2 | 1 | 5 |
| | Winter | 1 | 1 | 1 | 1 | 4 |
| T4 | Spring | - | - | - | - | 0 |
| | Summer | - | - | - | - | 0 |
| | Autumn | 1 | 1 | 2 | 2 | 6 |
| | Winter | 1 | 1 | 1 | 1 | 4 |
| T5 | Spring | 1 | 1 | 1 | 1 | 4 |
| | Summer | 1 | 1 | 0 | 1 | 3 |
| | Autumn | 1 | 1 | 4 | 2 | 8 |
| | Winter | - | - | - | - | 0 |
| T6 | Spring | 1 | 1 | 1 | 1 | 4 |
| | Summer | 1 | 1 | 1 | 3 | 6 |
| | Autumn | 1 | 1 | 0 | 4 | 6 |
| | Winter | 1 | 1 | 2 | 3 | 7 |
| T7 | Spring | 1 | 1 | 0 | 2 | 4 |
| | Summer | 1 | 1 | 0 | 1 | 3 |
| | Autumn | 1 | 1 | 0 | 2 | 4 |
| | Winter | 1 | 1 | 0 | 2 | 4 |

M = male, F = female

**Supplementary Table S11.** LMM investigating the effects of social status and the interaction between sex and season on network strength, calculated from whole days, for residents in networks with non-random associations: reduced model results after non-significant fixed effects in the full model were removed. $P_{rand}$ is the proportion of model coefficients based on randomised data that were greater than those based on observed data. Significant $P_{rand}$ values are shown in bold when 0.025 > $P_{rand}$ > 0.975. Unit of analysis = strength. N = 134 observations.

| **Model parameter** | **β** | **SE** | **df** | **t** | **$P_{rand}$** |
|---|---|---|---|---|---|
| *Fixed effects* | | | | | |
| Intercept | 1.456 | 0.171 | 96 | 8.522 | 0.889 |
| Social status (subordinate) | -0.090 | 0.137 | 42 | -0.662 | **1.000** |
| Sex (male) | 0.063 | 0.205 | 129 | 0.309 | **0.982** |
| Season (summer) | 0.027 | 0.188 | 95 | 0.146 | 0.733 |
| Season (autumn) | -0.194 | 0.168 | 94 | -1.152 | **0.995** |
| Season (winter) | -0.291 | 0.173 | 93 | -1.680 | 0.896 |
| Sex (male) : season (summer) | 0.072 | 0.287 | 98 | 0.250 | 0.034 |
| Sex (male) : season (autumn) | 0.205 | 0.240 | 97 | 0.853 | **0.013** |
| Sex (male) : season (winter) | -0.306 | 0.249 | 95 | -1.230 | 0.741 |
| | | | | | |
| *Random effects* | *Variance* | *SD* | *% total* | | |
| Individual ID (N = 51) | 0.102 | 0.319 | 29 | | |
| Territory (N = 7) | 0.000 | 0.000 | 0 | | |
| Residual | 0.249 | 0.499 | 71 | | |

Reference categories were social status = dominant, sex = female and season = spring

**Supplementary Table S12.** LMM investigating the effects of sex, status and their interactions with season on strength, calculated from whole days, for resident foxes in networks with non-random associations: full model results. $P_{rand}$ is the proportion of model coefficients based on randomised data that were greater than those based on observed data. Significant $P_{rand}$ values are shown in bold when 0.025 > $P_{rand}$ > 0.975. Unit of analysis = strength. N = 134 observations.

| **Model parameter** | **β** | **SE** | **df** | **t** | **$P_{rand}$** |
|---|---|---|---|---|---|
| *Fixed effects* | | | | | |
| Intercept | 1.525 | 0.197 | 123 | 7.722 | **<0.001** |
| Sex (male) | 0.065 | 0.205 | 129 | 0.320 | **0.983** |
| Season (summer) | -0.083 | 0.258 | 90 | -0.323 | 0.692 |
| Season (autumn) | -0.324 | 0.230 | 90 | -1.407 | **0.999** |
| Season (winter) | -0.325 | 0.241 | 91 | -1.349 | 0.883 |
| Social status (subordinate) | -0.204 | 0.212 | 126 | -0.963 | **0.993** |
| Sex (male) : season (summer) | 0.082 | 0.288 | 97 | 0.286 | 0.043 |
| Sex (male) : season (autumn) | 0.205 | 0.239 | 97 | 0.858 | **0.011** |
| Sex (male) : season (winter) | -0.309 | 0.248 | 94 | -1.250 | 0.735 |
| Social status (subordinate) : season (summer) | 0.181 | 0.288 | 95 | 0.631 | 0.464 |
| Social status (subordinate) : season (autumn) | 0.205 | 0.248 | 93 | 0.828 | 0.064 |
| Social status (subordinate) : season (winter) | 0.060 | 0.258 | 92 | 0.231 | 0.305 |
| | | | | | |
| *Random effects* | *Variance* | *SD* | *% total* | | |
| Individual ID (N = 51) | 0.103 | 0.321 | 30 | | |
| Territory (N = 7) | 0.000 | 0.000 | 0 | | |
| Residual | 0.247 | 0.497 | 70 | | |

Reference categories were sex = female, season = spring and social status = dominant

**Supplementary Table S13.** LMM investigating the interaction effect of sex and season on local weighted clustering coefficient for resident foxes in networks with non-random associations: reduced model results after non-significant fixed effects in the full model were removed. $P_{rand}$ is the proportion of model coefficients based on randomised data that were greater than those based on observed data. Significant $P_{rand}$ values are shown in bold when $0.025 > P_{rand} > 0.975$. Unit of analysis = local weighted clustering coefficient. N = 133 observations.

| **Model parameter** | **β** | **SE** | **df** | **t** | **$P_{rand}$** |
|---|---|---|---|---|---|
| *Fixed effects* | | | | | |
| Intercept | 0.287 | 0.044 | 15 | 6.473 | **<0.001** |
| Sex (male) | 0.031 | 0.039 | 125 | 0.787 | **0.006** |
| Season (summer) | 0.012 | 0.040 | 100 | 0.300 | 0.644 |
| Season (autumn) | -0.023 | 0.036 | 99 | -0.636 | 0.289 |
| Season (winter) | -0.115 | 0.037 | 97 | -3.133 | **0.991** |
| Sex (male) : season (summer) | 0.023 | 0.060 | 105 | 0.384 | 0.630 |
| Sex (male) : season (autumn) | -0.045 | 0.050 | 103 | -0.888 | **1.000** |
| Sex (male) : season (winter) | 0.004 | 0.052 | 101 | 0.071 | **0.900** |
| | | | | | |
| *Random effects* | *Variance* | *SD* | *% total* | | |
| Individual ID (N = 51) | 0.0003 | 0.022 | 2 | | |
| Territory (N = 7) | 0.008 | 0.092 | 42 | | |
| Residual | 0.011 | 0.106 | 56 | | |

Reference categories were sex = female and season = spring

**Supplementary Table S14.** Sex and seasonal differences in local clustering coefficient for resident foxes in networks with non-random associations. $P_{rand}$ is the proportion of model coefficients based on randomised data that were greater than those based on observed data. Significant $P_{rand}$ values are shown in bold when $0.025 > P_{rand} > 0.975$. Holm-adjusted $P_{rand}$ values are one-tailed so are considered significant when $P_{rand} < 0.05$.

| Sex | Season | Contrast | Estimated difference | SE | df | t-ratio | $P_{rand}$ | Holm-adj. $P_{rand}$ |
|---|---|---|---|---|---|---|---|---|
| M | - | SP- SU | -0.035 | 0.047 | 114 | -0.746 | 0.177 | 0.765 |
| M | - | SP - AU | 0.067 | 0.038 | 110 | 1.783 | **<0.001** | **0.001** |
| M | - | SP - WI | 0.111 | 0.039 | 107 | 2.846 | **<0.001** | **0.001** |
| M | - | SU - AU | 0.102 | 0.046 | 126 | 2.217 | **0.003** | **0.039** |
| M | - | SU - WI | 0.146 | 0.047 | 123 | 3.103 | **0.007** | 0.077 |
| M | - | AU - WI | 0.043 | 0.037 | 109 | 1.162 | 0.638 | 0.867 |
| F | - | SP- SU | -0.012 | 0.041 | 106 | -0.289 | 0.357 | 0.867 |
| F | - | SP - AU | 0.023 | 0.037 | 104 | 0.614 | 0.712 | 0.867 |
| F | - | SP - WI | 0.115 | 0.038 | 103 | 3.027 | **0.010** | 0.100 |
| F | - | SU - AU | 0.035 | 0.038 | 107 | 0.896 | 0.847 | 0.765 |
| F | - | SU - WI | 0.127 | 0.040 | 112 | 3.179 | 0.066 | 0.406 |
| F | - | AU - WI | 0.092 | 0.035 | 103 | 2.639 | **<0.001** | **0.001** |
| - | SP | F-M | -0.031 | 0.040 | 132 | -0.761 | **0.995** | **0.060** |
| - | SU | F-M | -0.054 | 0.048 | 133 | -1.106 | **0.983** | 0.136 |
| - | AU | F-M | 0.014 | 0.035 | 132 | 0.400 | **0.010** | 0.100 |
| - | WI | F-M | -0.034 | 0.038 | 132 | -0.911 | 0.943 | 0.406 |

M = male, F = female, SP = spring, SU = summer, AU = autumn, WI = winter

**Supplementary Table S15.** Intra-class correlation coefficients (ICC) of centrality measures to show the repeatability of individual network position (N = 53, as all 28 networks were used) across seasons. Clustering coefficients were only calculated for individuals connected to at least two associates so NAs were excluded in the calculation of ICC. $P_{rand}$ is the proportion of ICCs based on randomised data that were greater than those based on observed data. Significant $P_{rand}$ values are shown in bold when $0.025 > P_{rand} > 0.975$.

| Centrality measure | Mean seasons observed per individual | Observed ICC | Mean permuted ICC | $P_{rand}$ |
|---|---|---|---|---|
| Strength | 2.857 | 0.206 | 0.209 | 0.541 |
| Eigenvector centrality | 2.857 | 0.349 | 0.315 | 0.160 |
| Clustering coefficient | 2.838 | 0 | 0.0001 | 0.231 |

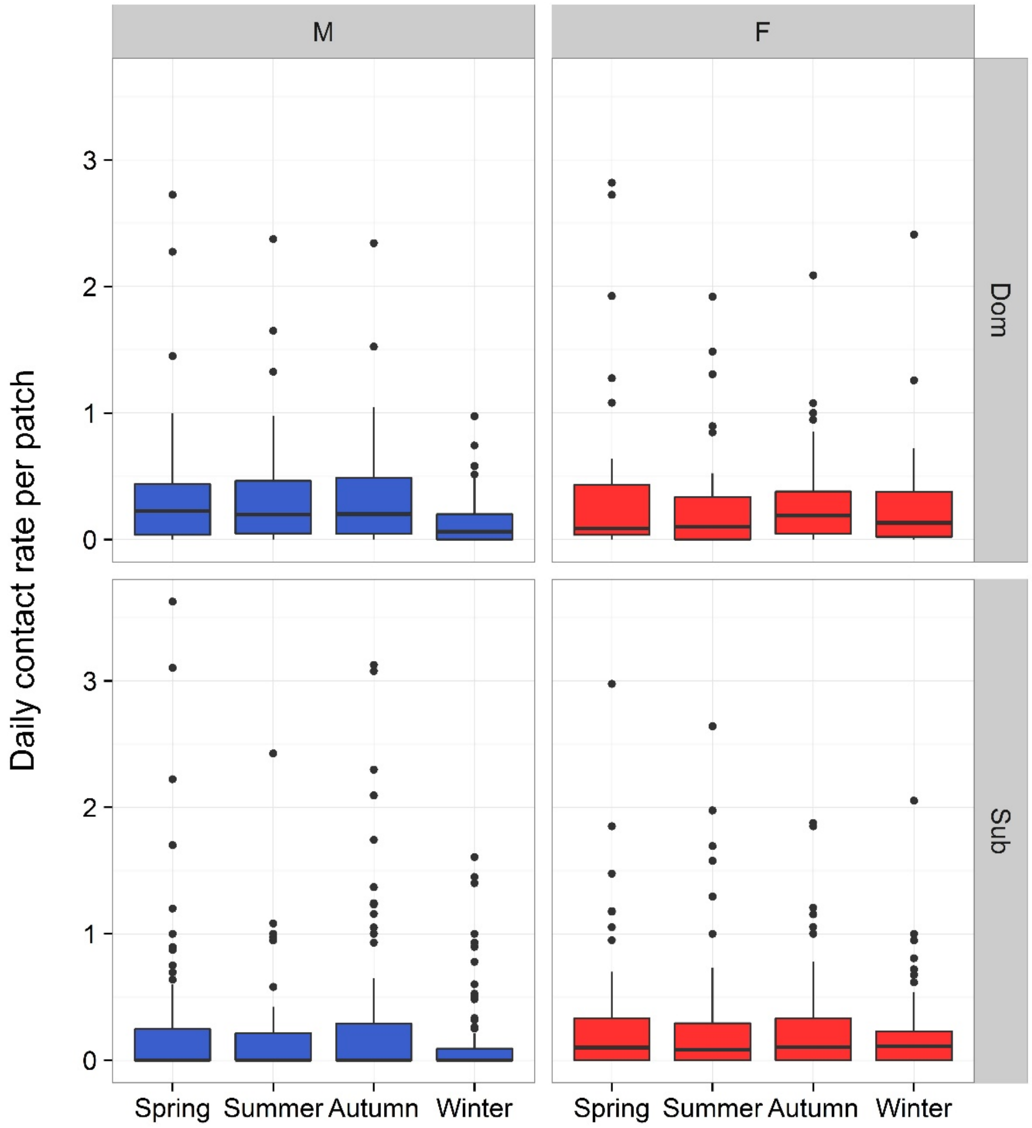


**Supplementary Figure S1.** Daily contact rates at food patches for foxes seen on ≥ 5 days in any territory (N = 132 individual foxes). Box plots show the median and 25th and 75th percentiles, whiskers indicate values within 1.5 times the interquartile range from these percentiles, and dots show values greater than 1.5 times the interquartile range from the 75th percentile. M = male, F = female, Dom = dominant, Sub = subordinate.

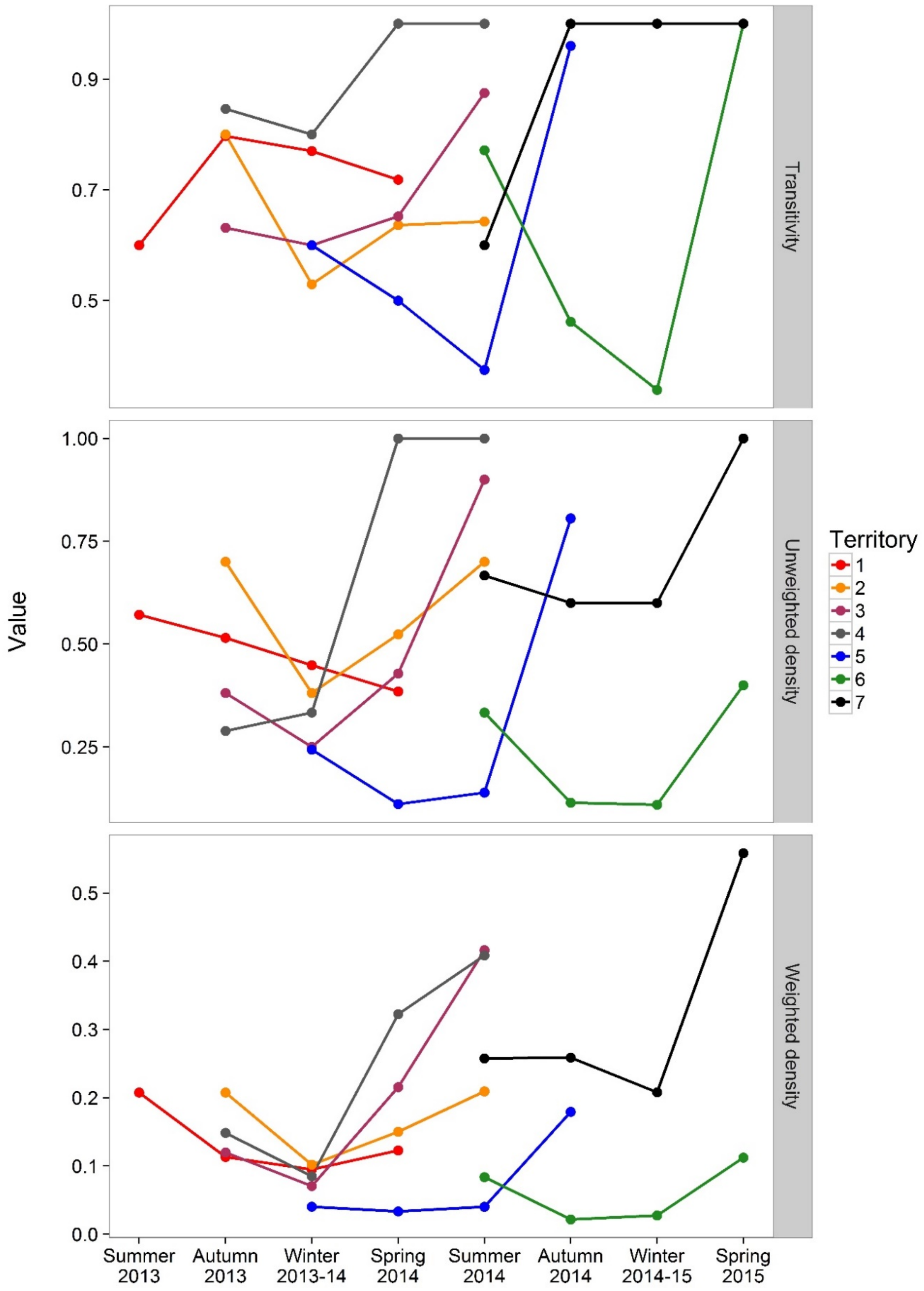


**Supplementary Figure S2.** Seasonal variation in transitivity, unweighted density and weighted density for the network in each territory, arranged in the order of data collection. Note that the y-axis is different for each plot.

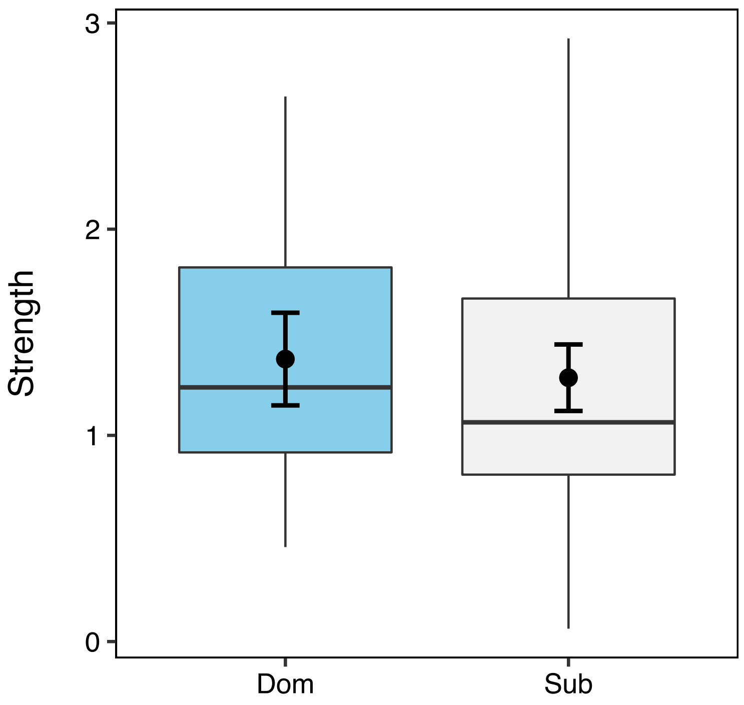


**Supplementary Figure S3.** Difference in network strength between dominant and subordinate resident foxes in networks with non-random associations. N = 51 individual foxes. Box plots show the raw data, with the median and 25th and 75th percentiles; whiskers indicate values within 1.5 times the interquartile range from these percentiles. Points are model predictions with 95% confidence intervals shown as error bars. Dom = dominant, Sub = subordinate.
